# Age and sex-specific stroke epidemiology in COVID-19

**DOI:** 10.3389/fstro.2023.1172854

**Published:** 2023-06-07

**Authors:** Youngran Kim, Maria A. Parekh, Xiaojin Li, Yan Huang, Guo-Qiang Zhang, Bharti Manwani

**Affiliations:** ^1^Department of Management, Policy and Community Health, School of Public Health, University of Texas Health Science at Houston, Houston, TX, United States; ^2^Department of Neurology, University of Texas Health Science at Houston, Houston, TX, United States

**Keywords:** COVID-19, stroke, epidemiology, sex differences, sex-specific stroke incidence

## Abstract

**Background:**

COVID-19 has emerged as an independent risk factor for stroke. We aimed to determine age and sex-specific stroke incidence and risk factors with COVID-19 in the US using a large electronic health record (EHR) that included both inpatients and outpatients.

**Methods:**

A retrospective cohort study was conducted using individual-level data from Optum^®^ de-identified COVID-19 EHR. A total of 387,330 individuals aged ≥ 18 with laboratory-confirmed COVID-19 between March 1, 2020 and December 31, 2020 were included. The primary outcome was cumulative incidence of stroke after COVID-19 confirmation within 180 days of follow-up or until death. Kaplan–Meier cumulative incidence curves for acute ischemic stroke (AIS), intracerebral hemorrhage (ICH), and a composite outcome of all strokes were stratified by sex and age, and the differences in curves were assessed using a log-rank test. The relative risk of stroke by demographics and risk factors was estimated using multivariable Cox-proportional hazards regressions and adjusted hazard ratios (aHRs).

**Results:**

Of 387,330 COVID-19 patients, 2,752 patients (0.71%, 95% CI 0.68–0.74) developed stroke during the 180-day follow-up, AIS in 0.65% (95% CI 0.62–0.67), and ICH in 0.11% (95% CI 0.10–0.12). Of strokes among COVID-19 patients, 57% occurred within 3 days. Advanced age was associated with a substantially higher stroke risk, with aHR 6.92 (5.72–8.38) for ages 65–74, 9.42 (7.74–11.47) for ages 75–84, and 11.35 (9.20–14.00) for ages 85 and older compared to ages 18–44 years. Men had a 32% higher risk of stroke compared to women. African-American [aHR 1.78 (1.61–1.97)] and Hispanic patients [aHR 1.48 (1.30–1.69)] with COVID-19 had an increased risk of stroke compared to white patients.

**Conclusion:**

This study has several important findings. AIS and ICH risk in patients with COVID-19 is highest in the first 3 days of COVID-19 positivity; this risk decreases with time. The incidence of stroke in patients with COVID-19 (both inpatient and outpatient) is 0.65% for AIS and 0.11% for ICH during the 180-day follow-up. Traditional stroke risk factors increase the risk of stroke in patients with COVID-19. Male sex is an independent risk factor for stroke in COVID-19 patients across all age groups. African-American and Hispanic patients have a higher risk of stroke from COVID-19.

## Introduction

COVID-19 caused by SARS-CoV-2 has been associated with increased cardiovascular disease, including myocardial infarction, myocarditis, acute ischemic stroke (AIS), and intracerebral hemorrhage (ICH) (D'Anna et al., 2020; Yaghi et al., 2020; Zubair et al., 2020). ICH has been a rare occurrence among hospitalized COVID-19 patients but is associated with higher mortality (Leasure et al., 2021). AIS is the most common stroke type in COVID-19 patients. Several studies have reported that AIS in COVID-19 patients is more likely to be due to large vessel occlusion (Kim et al., 2021; Nannoni et al., 2021), which is mostly attributable to systemic hypercoagulability caused by SARS-CoV-2 (Zhang et al., 2021). The incidence of AIS has been reported to be higher during the first few days to 2 weeks of COVID-19 diagnosis (Modin et al., 2020; Yang et al., 2022). Age and biological sex are critical determinants of health and disease, and they have a complex and interactive effect on stroke risk and pathophysiology. Sex differences in stroke have been well established with higher stroke incidence in men, but higher case fatality and worse functional outcomes in women (Appelros et al., 2009). Similarly, sex differences are implicated in the severity of COVID-19, with men more likely to exhibit enhanced disease severity and mortality than women (Scully et al., 2020; Takahashi et al., 2020). However, age and sex differences in stroke risk have not been well studied in patients with COVID-19. In this study, we estimated sex- and age-specific ischemic and hemorrhagic stroke risk in COVID-19 patients with a 6-month follow-up using a large electronic health record dataset.

## Materials and methods

The study protocol was reviewed and approved by the Institutional Review Board at The UTHealth Houston. This cohort study followed the Strengthening the Reporting of Observational Studies in Epidemiology (STROBE) reporting guidelines.

### Data source

We conducted a retrospective cohort study using Optum^®^ de-identified COVID-19 Electronic Health Record (EHR) data, which is sourced from Optum^®^'s longitudinal EHR repository derived from more than 700 hospitals and 7,000 clinics in the United States for inpatient and ambulatory electronic medical records. The dataset has been updated with a minimal time lag while preserving as much clinical information as possible, including new, unmapped COVID-specific clinical data points. At the time of our study, this dataset included 6.96 million unique individuals and contained patient-level, longitudinal clinical records including demographics, diagnoses, procedures, lab tests, care settings, and medications prescribed and/or administered.

### Study participants

We included individuals with laboratory-confirmed COVID-19-positive polymerase chain reaction (PCR) from March 1, 2020 to December 31, 2020 (*n* = 424,463). Antigen tests or diagnosis codes were not used to avoid potential false positivity of home tests or inaccurate time of COVID-19 diagnosis date. PCR tests were identified using Logical Observation Identifiers Names and Codes (LOINC), Current Procedural Terminology (CPT) codes, and test names in the EHR text (Huang and Zhang, 2021). The COVID-19 diagnosis date was based on the collected sample date for the first positive PCR result. We excluded individuals who were younger than 18 years of age at the time of COVID-19 (*n* = 36,861) or had missing age or sex information (*n* = 272). Patients who did not disclose or had unknown ethnicity were pooled in the ethnicity cohort called other.

### Outcome measures and covariates

The primary outcome was cumulative incidence of stroke since the COVID-19 diagnosis date, with a follow-up of 180 days or until death. It is possible that some patients may have had SARS-CoV-2 infection prior to hospitalization, and the PCR on admission date may have been a confirmatory or repeat COVID-19 PCR performed in the hospital. Therefore, we allowed a 3-day window between the recorded date of the COVID-19 test and the date of the stroke. Stroke was defined as hospitalization with a diagnosis of stroke using the International Classification of Diseases, Ninth Revision, and Clinical Modification (ICD-9-CM) and ICD-10-CM codes (Supplementary Table 1) for AIS and ICH. Although ICD-9-CM codes were replaced with ICD-10-CM codes as of October 2015, ICD-9 codes were also used to identify some medical records still reported using ICD-9 codes in the dataset. The month and year-of-death information was sourced from the Social Security Administration Death Master Files, and we set the date of death as the last date of the month. Data were also extracted for co-morbidities including congestive heart failure (CHF), hypertension, coronary artery disease (CAD), atrial fibrillation (AF), hyperlipidemia, diabetes, obesity, smoking status, and history of stroke, and they were identified using ICD-9 and 10 CM codes (Supplementary Table 1).

### Statistical analysis

Descriptive statistics for differences in characteristics between COVID-19 patients with and without stroke were assessed using the chi-square tests for categorical variables and Wilcoxon rank-sum tests for numeric variables. Stroke incidence was estimated as the percentage of COVID-19 patients that had a diagnosis of stroke during the 180-day follow-up period, and a 95% confidence interval (CI) was reported. Estimates were stratified by sex and age for any type of stroke and for AIS and ICH separately. Cumulative incidence curves for stroke were plotted using the Kaplan–Meier approach stratified by stroke type, sex, and age, and differences in the curves were assessed using a log-rank test. The relative risk of stroke based on demographics and risk factors was estimated as hazard ratios (HR), and a multivariable Cox-proportional hazard regression analysis was conducted for adjusted HRs including age, sex, race/ethnicity, CHF, hypertension, CAD, AF, hyperlipidemia, diabetes, obesity, smoking status, and history of stroke. Significance levels were set at *p* < 0.05 for two-tailed tests, and all analyses were performed using STATA 16.0 (StataCorp, College Station, TX).

## Results

### Characteristics of COVID-19 patients with/without stroke

A total of 387,330 patients with COVID-19 were included in the study. Of these patients, 61,026 (15.8%) were hospitalized and 2,752 patients developed a stroke within 180 days of their COVID-19 diagnosis. The median age of all COVID-19 patients was 47 (IQR 32–61) years; patients with stroke were significantly older than those who did not have a stroke [71 (IQR 60–80) vs. 47 (32–61) years, *p* < 0.001] (Table 1). Women accounted for 55.0% of all COVID-19 patients in our study, but there were significantly more men in the COVID-19 stroke group compared to the non-stroke group (57.2 vs. 44.9%, *p* < 0.001). As compared to non-stroke COVID-19 patients, COVID-19 patients with stroke had higher traditional stroke risk factors. These included hypertension (81.8 vs. 28.6%, *p* < 0.001), hyperlipidemia (67.0 vs. 23.8%, *p* < 0.001), diabetes (49.5 vs. 14.7%, *p* < 0.001), AF (29.7 vs. 4.5%, *p* < 0.001), CHF (28.9 vs. 4.6%, *p* < 0.001), CAD (35.4 vs. 6.8%, *p* < 0.001), obesity (34.5 vs. 17.3%, *p* < 0.001), smoking (42.0 vs. 16.2%, *p* < 0.001), and a prior history of stroke (16.5 vs. 0.6%, *p* < 0.001). When stratified by age, the differences in comorbidities between COVID-19 patients with and without stroke remained significant (Supplementary Tables 2, 3).

**Table 1:** Demographics and clinical characteristics of COVID-19 patients with and without stroke.

	**All COVID-19 patients (*n* = 387,330)**	**COVID-19 patients without stroke (*n* = 384,578)**	**COVID-19 patients with stroke (*n* = 2,752)**	***p*** **-value**
Age, median (IQR)	47 (32–61)	47 (32–61)	71 (60–80)	<0.001
**Age group**, ***n*** **(%)**				
18–44	176,715 (45.6)	176,563 (45.9)	152 (5.5)	<0.001
45–54	67,339 (17.4)	67,076 (17.4)	263 (9.6)	
55–64	67,259 (17.4)	66,731 (17.4)	528 (19.2)	
65–74	41,165 (10.6)	40,430 (10.5)	735 (26.7)	
75–84	23,030 (5.9)	22,349 (5.8)	681 (24.7)	
85+	11,822 (3.1)	11,429 (3.0)	393 (14.3)	
**Sex**, ***n*** **(%)**				
Male	174,392 (45.0)	172,819 (44.9)	1,573 (57.2)	<0.001
Female	212,938 (55.0)	211,759 (55.1)	1,179 (42.8)	
**Race and ethnicity**, ***n*** **(%)**				
White	251,521 (64.9)	249,879 (65.0)	1,642 (59.7)	<0.001
African-American	47,184 (12.2)	46,623 (12.1)	561 (20.4)	
Hispanic	45,979 (11.9)	45,713 (11.9)	266 (9.7)	
Other/unknown	42,646 (11.0)	42,363 (11.0)	283 (10.3)	
**Comorbidities**, ***n*** **(%)**				
CHF	18,444 (4.8)	17,650 (4.6)	794 (28.9)	<0.001
Hypertension	112,291 (29.0)	110,041 (28.6)	2,250 (81.8)	<0.001
CAD	27,048 (7.0)	26,074 (6.8)	974 (35.4)	<0.001
Atrial fibrillation	18,292 (4.7)	17,474 (4.5)	818 (29.7)	<0.001
Hyperlipidemia	93,216 (24.1)	91,372 (23.8)	1,844 (67.0)	<0.001
Diabetes	57,769 (14.9)	56,406 (14.7)	1,363 (49.5)	<0.001
Obesity	67,477 (17.4)	66,528 (17.3)	949 (34.5)	<0.001
Smoking history	41,991 (10.8)	41,092 (10.7)	899 (32.7)	<0.001
Current smoker	26,917 (6.9)	26,537 (6.9)	380 (13.8)	<0.001
History of stroke	2,792 (0.7)	2,338 (0.6)	454 (16.5)	<0.001
AIS	2,581 (0.7)	2,151 (0.6)	430 (15.6)	<0.001
ICH	402 (0.1)	327 (0.1)	75 (2.7)	<0.001

### Age and sex differences in stroke incidence in COVID-19 patients

**Figure 1:**
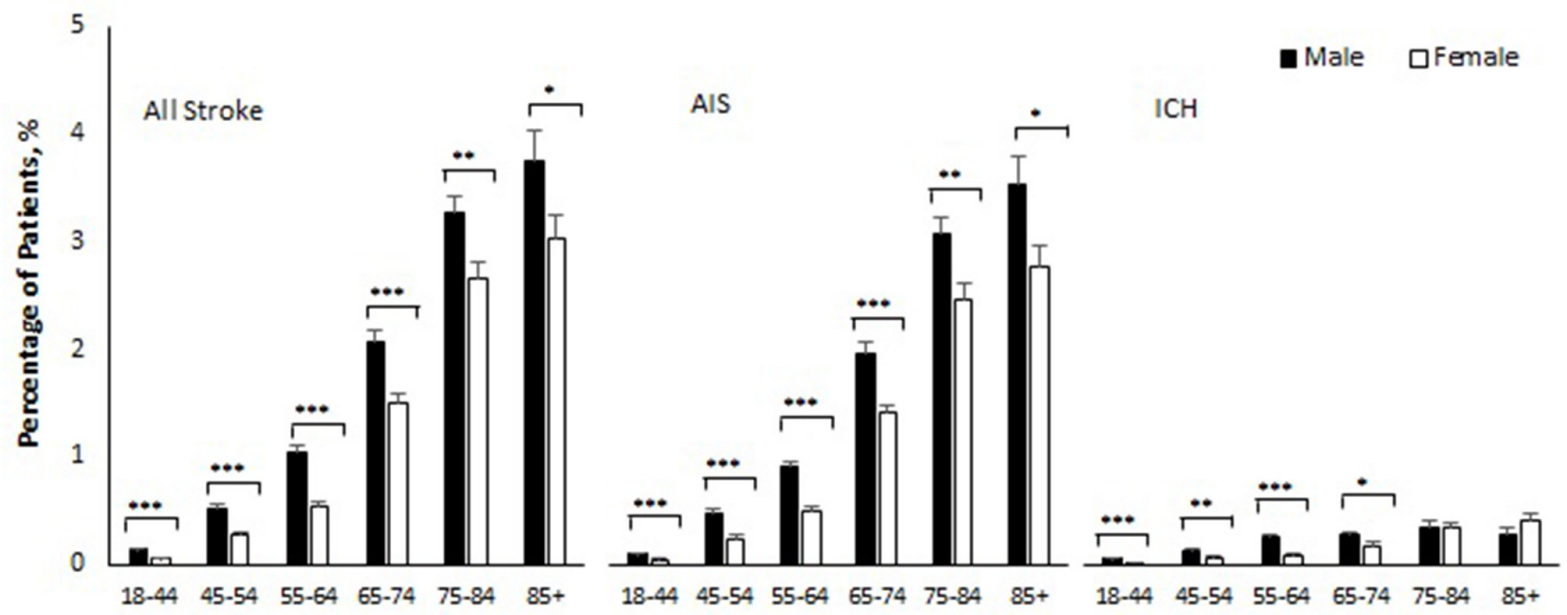
Percentage of patients who developed stroke within 180 days of COVID-19 by sex and age. AIS indicates acute ischemic stroke; ICH intracerebral hemorrhage. Error bars show standard errors of estimates. Age-specific-sex differences were all statistically significant (***p < 0.001, **p < 0.01, and *p < −0.05) except for ICH in age 75–84 (*p* = 0.98) and age 85 and older (*p* = 0.27).

Table 2 and Figure 1 show sex-specific stroke incidence within 180 days of COVID-19 by age and subtype of stroke. The overall stroke incidence within 180 days of COVID-19 diagnosis was 0.71% (95% CI 0.68–0.74), AIS 0.65% (95% CI 0.62–0.67), and ICH 0.11% (95% CI 0.10–0.12). Stroke incidence was higher in men as compared with women (0.90 vs. 0.55%, *p* < 0.001), for both AIS (0.82 vs. 0.51%, *p* < 0.001) and ICH (0.15 vs. 0.08%, *p* < 0.001). Age-stratified analysis showed that men with COVID-19 had a significantly higher incidence of AIS as compared with women across all age groups (Table 2). Similarly, men with COVID-19 had a higher incidence of ICH as compared to women in the age groups 18–44 (0.05 vs. 0.01%, *p* < 0.001), 45–54 (0.12 vs. 0.06%, *p* = 0.011), 55–64 (0.25 vs. 0.09%, *p* < 0.001), and 65–74 years (0.27 vs. 0.18%, *p* = 0.049). This sex difference, however, became insignificant in COVID-19 patients aged 75 or older and there was a nonsignificant reversal of incidence seen in COVID-19 patients at >85 years of age, with women having a trend toward higher incidence as compared to men (0.40 vs. 0.27%, *p* = 0.27) (Table 2).

**Table 2:** Sex-specific stroke incidence within 180 days of COVID-19 by age and subtype of stroke.

	**All**	**Male**	**Female**	***p*** **-value**
**Stroke, % (95% CI)**				
Overall	0.71 (0.68–0.74)	0.90 (0.86–0.95)	0.55 (0.52–0.59)	<0.001
**By age**				
18–44	0.09 (0.07–0.10)	0.14 (0.11–0.17)	0.05 (0.04–0.07)	<0.001
45–54	0.39 (0.35–0.44)	0.52 (0.44–0.60)	0.28 (0.23–0.34)	<0.001
55–64	0.79 (0.72–0.85)	1.04 (0.94–1.16)	0.54 (0.47–0.63)	<0.001
65–74	1.79 (1.66–1.92)	2.07 (1.89–2.28)	1.50 (1.34–1.67)	<0.001
75–84	2.96 (2.75–3.18)	3.26 (2.95–3.61)	2.67 (2.39–2.97)	0.008
85 and older	3.32 (3.02–3.66)	3.75 (3.25–4.33)	3.04 (2.66–3.46)	0.033
**AIS, % (95% CI)**				
Overall	0.65 (0.62–0.67)	0.82 (0.78–0.86)	0.51 (0.48–0.54)	<0.001
**By age**				
18–44	0.07 (0.06–0.08)	0.10 (0.08–0.13)	0.04 (0.03–0.06)	<0.001
45–54	0.35 (0.30–0.39)	0.47 (0.40–0.55)	0.24 (0.19–0.30)	<0.001
55–64	0.69 (0.63–0.76)	0.91 (0.81–1.02)	0.49 (0.42–0.57)	<0.001
65–74	1.68 (1.56–1.81)	1.96 (1.78–2.16)	1.40 (1.25–1.57)	<0.001
75–84	2.76 (2.55–2.98)	3.07 (2.76–3.40)	2.47 (2.20–2.76)	0.005
>85	3.07 (2.77–3.40)	3.52 (3.03–4.09)	2.77 (2.41–3.18)	0.020
**ICH, % (95% CI)**				
Overall	0.11 (0.10–0.12)	0.15 (0.13–0.17)	0.08 (0.07–0.09)	<0.001
**By age**				
18–44	0.03 (0.02–0.04)	0.05 (0.04–0.07)	0.01 (0.01–0.02)	<0.001
45–54	0.09 (0.07–0.11)	0.12 (0.09–0.16)	0.06 (0.04–0.09)	0.011
55–64	0.17 (0.14–0.20)	0.25 (0.20–0.31)	0.09 (0.06–0.13)	<0.001
65–74	0.22 (0.18–0.27)	0.27 (0.20–0.35)	0.18 (0.13–0.24)	0.049
75–84	0.34 (0.27–0.42)	0.34 (0.25–0.47)	0.34 (0.25–0.46)	0.98
>85	0.35 (0.26 −0.47)	0.27 (0.16–0.47)	0.40 (0.27–0.57)	0.27

**Figure 2:**
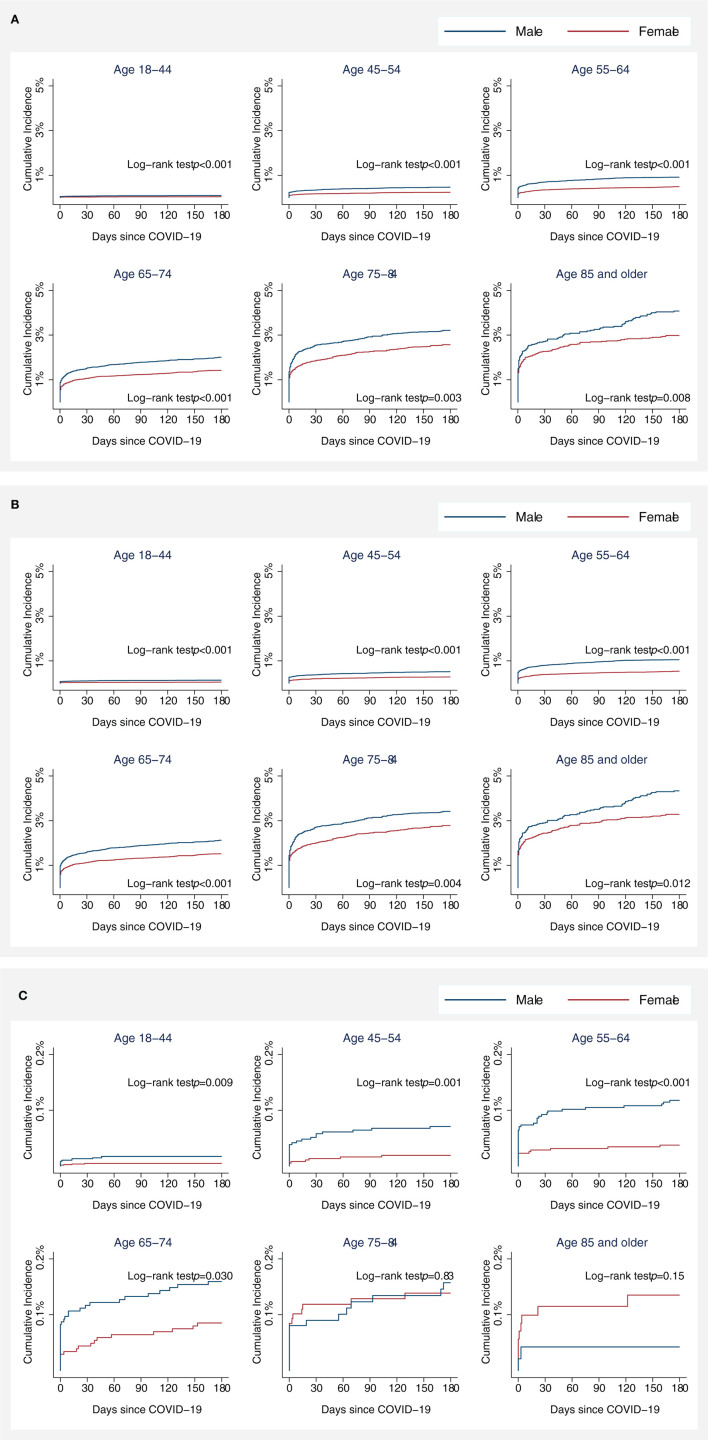
Kaplan–Meier (KM) cumulative incidence curve for all stroke **(A)**, acute ischemic stroke **(B)**, and intracranial hemorrhage **(C)** among COVID-19 patients by sex and age. Cumulative incidences of acute ischemic stroke were plotted since the date of COVID-19 confirmation using KM estimates. Statistical differences in KM curves between male and female patients were assessed using a log-rank test.

The Kaplan–Meier cumulative incidence curves for stroke showed that approximately half of the strokes among COVID-19 patients occurred early in the follow-up time, regardless of sex and age (Figure 2A), 57% of the strokes within 3 days of COVID-19 laboratory confirmation, 63% within 7 days, 70% within 14 days, 73% within 21 days, and 76% within 28 days. Age-specific cumulative incidence remained greater in men compared to women during follow-up. These patterns were observed for AIS and ICH separately as well, except for ICH patients aged 75 and older (Figures 2B, C).

### Factors associated with stroke in COVID-19 patients

The risk of stroke increased substantially with age among COVID-19 patients. Sex and comorbidity aHRs were 2.81 (95%CI 2.29–3.45) for ages 45–54, 4.16 (95% CI 3.43–5.04) for ages 55–64, 6.92 (95% CI 5.72–8.38) for ages 65–74, 9.42 (95% CI 7.74–11.47) for ages 75–84, and 11.35 (95% CI 9.20–14.00) for ages 85 and older, as compared to ages 18–44 years (Table 3). Risks for AIS and ICH increased with older age as well. Men with COVID-19 had a 32% higher risk of stroke compared to women with COVID-19 [aHR 1.32 (95% CI 1.22–1.43)]. Compared to white patients, African-American [aHR 1.78 (95% CI 1.61–1.97)] and Hispanic patients [aHR 1.48 (95% CI 1.30–1.69)] had increased risk of stroke, even after adjusting for sex and comorbid conditions (Table 3). These sex and racial/ethnic differences in stroke risk were observed for AIS and ICH as well.

**Table 3:** Relative risk of all stroke, AIS, and ICH among COVID-19 patients^*^.

	**All Stroke**		**AIS**		**ICH**	
	**HR (95% CI)**	***p*** **-value**	**HR (95% CI)**	***p*** **-value**	**HR (95% CI)**	***p*** **-value**
**Age**						
18–44	1.00 (Reference)		1.00 (Reference)		1.00 (Reference)	
45–54	2.81 (2.29–3.45)	<0.001	3.19 (2.54 −4.00)	<0.001	2.63 (1.43–4.83)	0.002
55–64	4.16 (3.43–5.04)	<0.001	4.65 (3.76–5.76)	<0.001	3.48 (1.94–6.24)	<0.001
65–74	6.92 (5.72–8.38)	<0.001	8.24 (6.67–10.18)	<0.001	3.94 (2.14–7.24)	<0.001
75–84	9.42 (7.74–11.47)	<0.001	11.03 (8.88–13.72)	<0.001	3.83 (1.97–7.44)	<0.001
>85	11.35 (9.20–14.00)	<0.001	13.19 (10.48–16.61)	<0.001	2.73 (1.19–6.26)	0.02
**Sex**						
Female	1.00 (Reference)		1.00 (Reference)		1.00 (Reference)	
Male	1.32 (1.22–1.43)	<0.001	1.31 (1.21–1.42)	<0.001	1.83 (1.35–2.48)	0.001
**Race/ethnicity**						
White	1.00 (Reference)		1.00 (Reference)		1.00 (Reference)	
African-American	1.78 (1.61–1.97)	<0.001	1.82 (1.65–2.02)	<0.001	2.33 (1.62–3.37)	<0.001
Hispanic	1.48 (1.30–1.69)	<0.001	1.39 (1.21–1.61)	<0.001	1.68 (1.03–2.75)	0.038
Other/unknown	1.83 (1.61–2.08)	<0.001	1.74 (1.52–2.00)	<0.001	3.28 (2.19–4.92)	<0.001
**Risk factors**						
CHF	1.20 (1.09–1.33)	<0.001	1.22 (1.10–1.36)	<0.001	1.29 (0.86–1.93)	0.22
Hypertension	2.52 (2.23–2.84)	<0.001	2.45 (2.16–2.79)	<0.001	2.55 (1.66–3.93)	<0.001
CAD	1.20 (1.10–1.32)	0.001	1.21 (1.10–1.33)	<0.001	0.96 (0.65–1.40)	0.82
AF	1.66 (1.51–1.83)	<0.001	1.63 (1.48–1.80)	<0.001	2.68 (1.83–3.92)	<0.001
Hyperlipidemia	1.20 (1.09–1.32)	0.003	1.25 (1.13–1.38)	<0.001	1.04 (0.73–1.49)	0.83
Diabetes	1.41 (1.29–1.53)	<0.001	1.44 (1.32–1.57)	<0.001	1.69 (1.21–2.37)	0.002
Obesity	1.18 (1.08–1.28)	0.001	1.14 (1.04–1.25)	0.003	1.23 (0.89–1.70)	0.22
Smoking	1.34 (1.24–1.46)	<0.001	1.35 (1.24–1.47)	<0.001	1.08 (0.78–1.49)	0.66
Stroke history	6.17 (5.54–6.88)	<0.001	6.10 (5.45–6.82)	<0.001	4.39 (2.73–7.06)	<0.001

^*^Relative risk of stroke based on demographics and risk factors was estimated as hazard ratios (HR) and multivariable Cox-proportional hazard regression was conducted for adjusted hazard ratios including age, sex, race/ethnicity, congestive heart failure (CHF), hypertension, coronary artery disease (CAD), atrial fibrillation (AF), hyperlipidemia, diabetes, obesity, smoking status, and history of stroke.

Co-morbid conditions that included CHF, hypertension, CAD, AF, hyperlipidemia, diabetes, obesity, prior history of stroke, and smoking were independently associated with increased risk of AIS in COVID-19 patients. Hypertension [aHR 2.55 (95%CI 1.66–3.93)], AF [aHR 2.68 (95% CI 1.83–3.92)], diabetes [aHR 1.69 (95% CI 1.21–2.37)], and prior history of stroke [aHR 4.39 (95% CI 2.73–7.06)] were independently associated with increased risk for ICH in COVID-19 patients.

## Discussion

This is one of the largest studies on stroke and COVID-19 and included 387,330 COVID-19 patients in the US. We found that stroke incidence rate within 6 months after COVID-19 was 0.71%, with AIS being 0.65% and ICH 0.11%. We confirmed that traditional stroke risk factors increase the risk of stroke even in COVID-19 patients. Aging and male sex are independent risk factors for stroke in COVID-19 patients. Men with COVID-19 have a 32% higher risk of stroke as compared to women, and this risk does not reverse at advanced age, unlike other stroke etiologies. Moreover, African-American and Hispanic patients with COVID-19 have an increased risk of stroke compared to white patients with COVID-19.

The reported incidence of stroke among COVID-19 patients has varied in prior studies depending on region, age, follow-up duration, and hospitalization. Our reported rates of 0.65% AIS is lower than some of the earlier studies (Li et al., 2020) but closer to estimates from a recent meta-analysis, which showed a pooled prevalence of AIS to be ~2% in COVID-19 patients, with a range of 0.9–4.6% for individual studies in another meta-analysis (Luo et al., 2022; Stefanou et al., 2023). A 1.6 and 3.7% overall stroke incidence has been reported in cohorts of hospitalized and critically ill COVID-19 patients, respectively (Bilaloglu et al., 2020). Stroke risk is higher in COVID-19 hospitalized patients, as compared to patients who recover from COVID-19 in the outpatient (Chavda et al., 2022). Our study included all COVID-19 patients, both inpatient and outpatient, which is a better representation of the population at large, but may have diluted the incidence rate (Qureshi et al., 2021) when compared to prior reported incidences only in hospitalized COVID-19 patients (Mao et al., 2020). Moreover, it is also important to understand that the reported incidences are for 6 months after COVID-19. For similar reasons, our ICH rate of 0.11% is also slightly lower than the reported ICH risk of 0.14–0.86% in patients hospitalized with COVID-19 (Margos et al., 2021). Although our study focused solely on stroke incidence in COVID-19 patients and did not include a comparison group of non-COVID-19 patients or the general population, our estimate of 0.71% for overall stroke (AIS and ICH) seems consistent with more recent reports (Qureshi et al., 2021).

We also report that 57% of strokes occur in the first 3 days of COVID-19 diagnosis and that stroke risk from COVID-19 decreases with time. Although this result comes with the caveat that we allowed a 3-day window between the recorded date of the COVID-19 test and the date of stroke, it still gives us an accurate estimate that the acute phase of COVID-19 is the high-risk period for stroke. The reported risk of stroke (both AIS and ICH) in the acute phase of COVID-19 is consistent with previous reports (Modin et al., 2020; Katsanos et al., 2021; Katsoularis et al., 2021; Yang et al., 2022). The increased risk of AIS and ICH in the acute phase of COVID-19, specifically in hospitalized patients with increased disease severity, is due to differing mechanisms. For AIS, the inflammatory storm and hypercoagulable state appear to be responsible for the thrombotic episodes (Qi and Huang, 2021; Luo et al., 2022). Other mechanisms that have been described are vasculitis and cardiomyopathy (Tsivgoulis et al., 2020). ICH in COVID-19 patients is presumably due to endothelial injury and inflammation caused by viral invasion (Benger et al., 2020; Margos et al., 2021), leading to the frailty of the vessel wall and hemorrhage. The use of anticoagulation for thrombosis prevention in patients with COVID-19 is also known to increase ICH risk (Melmed et al., 2021). We observed that all cardiovascular risk factors were independently associated with an increased risk of all types of stroke and AIS in patients with COVID-19 (Merkler et al., 2020; Scutelnic and Heldner, 2020; Qureshi et al., 2021). Hypertension, AF, diabetes, and a prior history of stroke were independently associated with an increased risk of ICH among COVID-19 stroke patients in our study. The risk of ICH with hypertension and prior history of stroke is well known (Brott et al., 1986). AF is treated with anticoagulation, and we believe that the higher risk of ICH seen in COVID-19 patients with AF may be related anticoagulant use, which has been observed in other studies (Margos et al., 2021). Diabetes mellitus and hyperglycemia are known to increase the risk of bleeding (Wang et al., 2020) but the complex interaction of SARS-CoV-2 infection with diabetes mellitus that leads to ICH remains unexplored and could be the focus of future studies.

Aging was associated with an increased risk of stroke. Some studies have observed that stroke patients with COVID-19 tend to be younger compared to COVID-19-negative stroke patients (Yaghi et al., 2020). The median age of stroke patients in our study was 71 years, which is comparable to the median age of AIS in the Multinational COVID-19 Stroke Study Group report (68 years) (Shahjouei et al., 2021), and in the multinational study on 174 AIS patients with SARS-CoV-2 infection (71 years) (Ntaios et al., 2020). Although our study did not compare the risk of stroke between COVID-19 and non-COVID-19 patients, our results indicate that patients with COVID-19 and stroke are significantly older and more likely to have co-existing cardiovascular risk factors when compared to COVID-19 non-stroke group.

We report sex differences in stroke from COVID-19 in all age groups. It is now well understood that biologic mechanisms of cell death in the ischemic brain are influenced by sex (Bushnell et al., 2018). Sex differences in coagulation, sex hormones, reproductive factors including pregnancy and childbirth, and social factors, all can influence the risk of stroke and impact stroke outcomes (Bushnell et al., 2014). Men with COVID-19 had a 32% higher risk for ischemic stroke compared to women, and age-specific cumulative incidence remained greater in men compared to women during the follow-up period (Finsterer et al., 2022). Age-specific sex differences were significant for both AIS and ICH in our cohort except for patients aged 75–84 and 85 and older in the ICH group (Figure 1). Historically, the age-adjusted incidence of stroke is higher in men compared to women (Carandang et al., 2006; Petrea et al., 2009), except in the elderly (Petrea et al., 2009) where with advanced age (>85 years of age) the incidence of stroke and stroke-related mortality and disability is higher in women (Petrea et al., 2009; Bushnell et al., 2014). Our retrospective analysis followed the age and sex stratification used in the Framingham heart study, which is one of the largest prospective studies (with 56 years follow-up) of sex differences in stroke (Petrea et al., 2009). It was interesting to see that unlike stroke caused by other risk factors, the risk of stroke from COVID-19 remains higher in men throughout the lifespan. This is a novel and important finding as it highlights that COVID-19-related stroke may be caused by enhanced inflammation/cytokine storm, which is known to be higher in men who get infected with COVID-19, as compared to women (Gebhard et al., 2020).

While the effect of sex on AIS is well known (Reeves et al., 2008; Appelros et al., 2009), sex differences in ICH incidence are less clear, with most studies reporting no effect of sex (D'Alessandro et al., 2000), and others reporting higher incidence in men (Thrift et al., 2009; Gokhale et al., 2015). In our cohort of COVID-19 patients, men were observed to have an 83% higher risk of ICH compared to female patients. However, sex differences in ICH were not statistically significant among patients aged 75 years or older, although there was a trend for incidence reversal among patients aged 85 years or older, which did not reach statistical significance (0.40 vs. 0.27%, *p* = 0.27). This was likely due to the low number of elderly male patients and the rarity of ICH cases. Specifically, there were only 4,742 men compared to 7,080 women in the ≥85 years age group. We believe that the low sample size (only 41 cases of ICH) in that age group did not provide statistical power to distinguish any sex differences even if they existed. A prior study in Southeast Asian ICH patients (Hsieh et al., 2016) has shown increased ICH in women aged 80 years or older (21.5 vs. 9.5%, *p* < 0.001). We postulate that increased anticoagulation use in women of advanced age may be the underlying cause for this trend. Future studies to explore other underlying mechanisms are needed.

We also observed ethnic and racial disparities in COVID-19 and stroke, with African-American and Hispanic patients at 78 and 48% increased risk for stroke, respectively, compared with white patients. Prior studies have reported a 2–3-fold increased risk of stroke and associated mortality among African-American and Hispanic people in the US, and this is attributed to a higher prevalence of cardiovascular risk factors in this population (Howard et al., 2011; Gardener et al., 2020). The underlying causes of health disparities are complex and include social and structural determinants of health, discrimination, economic and educational disadvantages, health care access and quality, and individual behavior and biology, including the disproportionate risk of underlying comorbidities in different racial and ethnic minority populations. The COVID-19 pandemic has served to yet again emphasize these disturbing health disparities (Webb Hooper and Pérez-Stable, 2020). Understanding the social determinants among these high-risk populations is critical for inclusivity and for the formulation of strategies to address the fundamental causes of disparities and design social determinant-focused interventions in patients with COVID-19.

In summary, in this large national cohort study of adults aged 18 years and older with COVID-19 in the US, we observed that male sex, advanced age, and being an African-American or Hispanic with COVID-19, all increase the risk of stroke. In COVID-19 patients, age-specific cumulative incidence for AIS is greater in men compared to women during the follow-up period of 180 days in all stroke and AIS across all age groups, and in ICH for patients aged 18–74 years (with a nonsignificant trend toward incidence reversal in ≥75 years old). While prevention and management of stroke risk factors are important across all racial/ethnic and sex groups affected by COVID-19, it is important to understand the modifiable and non-modifiable risk factors for COVID-19-related stroke. This may allow the scientific, public health and clinical communities to identify the high-risk groups for stroke in COVID-19 and implement preventative strategies.

The work presented in this study is subject to several limitations, mainly due to the retrospective study design using secondary data. The diagnosis of stroke, AIS, ICH, and risk factors relied on hospital ICD diagnosis codes from a database, and verification of accurate diagnosis cannot be undertaken. It is important to note that a large number of patients (*n* = 42,646, 11.0%) had reported other or unknown ethnicity, which could lead to sampling bias and limit the generalizability of the results regarding race/ethnic differences in stroke risk due to COVID-19. Additionally, patient-level data such as stroke severity and etiology, acute interventions, management or treatments used, clinical outcomes, and socioeconomic status were not collected as they were not the focus of this study. Like any other study, our data may underestimate the true rates of concomitant SARS-CoV2 infection with a stroke diagnosis, depending on the frequency of testing at each site and across the study period. Moreover, since this study included patients from the beginning of the pandemic in March 2020, it may not define stroke risk with different SARS-CoV-2 subvariants and vaccination status.

## Data availability statement

The data analyzed in this study was obtained from Optum by a licensed agreement. Requests to access these datasets should be directed to https://www.optum.com.

## Ethics statement

The study protocol was reviewed and approved by the Committee for the Protection of Human Subjects (CPHS) at the University of Texas Health Science Center at Houston. Written informed consent for participation was not required for this study in accordance with the national legislation and the institutional requirements.

## Author contributions

MP, YK, XL, and BM contributed to the conception and design of the study. YK and XL organized the database. YK and YH performed the statistical analysis. MP, YK, and BM wrote the manuscript. All authors contributed to the revision of the manuscript, read, and approved the submitted version.
