# Supplementary material for: Age and sex-specific stroke epidemiology in COVID-19
Source: Front Stroke. 2023 Jun 7;2:1172854. doi: 10.3389/fstro.2023.1172854 (PMC11600532; doi:10.3389/fstro.2023.1172854)
Supplement: Supplementary file 1 [file Table_1.DOCX]

**Supplemental Table 1. International Classification of Diseases (ICD) Codes to Identify Disease Conditions**

| **Conditions** | **ICD-9 codes** | **ICD-10 codes** |
| --- | --- | --- |
| Ischemic stroke | 433.x1, 434.x1 | I63.x |
| Intracerebral hemorrhage | 431 | I61.x |
| Congestive heart failure | 398.91, 402.01, 402.11, 402.91, 404.01, 404.03, 404.11, 404.13, 404.91, 404.93, 428.x | I09.81, I11.0, I13.0, I13.2, I50.x |
| Hypertension | 401.xx-405.x | I10.x–I13.x, I15.x |
| Coronary artery disease | 414.01, 414.2, 414.3, 414.4 | I25.1x, I252, I25.82, I25.83, I25.84 |
| Atrial fibrillation | 427.31 | I48.0, I48.1x, I48.2x, I48.91 |
| Hyperlipidemia | 272.0, 272.1, 272.2, 272.3, 272.4 | E78.0x -E78.5x |
| Diabetes | 250.xx | E10.x, E11.x, E13.x |
| Obesity | 278.0x | E66.x |
| Smoking | 305.1, V15.82 | F17.x, Z72.0, Z87.891 |

**Supplemental Table 2. Characteristics of COVID-19 Patients with and without Stroke, Age <65 years**

|  | **Age 18-44** | | **Age 45-54** | | **Age 55-64** | |
| --- | --- | --- | --- | --- | --- | --- |
|  | No Stroke  (n=176,563) | Stroke (n=152) | No Stroke  (n=67,076) | Stroke (n=263) | No Stroke  (n=66,731) | Stroke (n=528) |
| **Sex, n (%)** |  |  |  |  |  |  |
| Male | 74,101 (42.0) | 101 (66.4) | 30,894 (46.1) | 161 (61.2) | 32,235 (48.3) | 340 (64.4) |
| Female | 102,462 (58.0) | 51 (33.6) | 36,182 (53.9) | 102 (38.8) | 34,496 (51.7) | 188 (35.6) |
| **Comorbidities, n (%)** | |  |  |  |  |  |
| CHF | 773 (0.4) | 19 (12.5) | 1,471 (2.2) | 72 (27.4) | 3,292 (4.9) | 117 (22.2) |
| Hypertension | 13,675 (7.7) | 72 (47.4) | 19,253 (28.7) | 189 (71.9) | 29,333 (44.0) | 419 (79.4) |
| CAD | 681 (0.4) | 15 (9.9) | 2,261 (3.4) | 56 (21.3) | 5,807 (8.7) | 167 (31.6) |
| AF | 486 (0.3) | 9 (5.9) | 1,043 (1.6) | 41 (15.6) | 2,911 (4.4) | 92 (17.4) |
| Hyperlipidemia | 9,141 (5.2) | 36 (23.7) | 15,663 (23.4) | 137 (52.1) | 25,743 (38.6) | 339 (64.2) |
| Diabetes | 7,254 (4.1) | 41 (27.0) | 9,986 (14.9) | 134 (51.0) | 15,117 (22.7) | 294 (55.7) |
| Obesity | 21,115 (12.0) | 64 (42.1) | 13,973 (20.8) | 111 (42.2) | 15,427 (23.1) | 238 (45.1) |
| Smoking | 18,660 (10.6) | 50 (32.9) | 9,311 (13.9) | 96 (36.5) | 13,015 (19.5) | 216 (40.9) |
| Hx of smoking | 8,308 (4.7) | 21 (13.8) | 5,339 (8.0) | 59 (22.4) | 9,011 (13.5) | 131 (24.8) |
| Current | 11,917 (6.7) | 35 (23.0) | 4,720 (7.0) | 55 (20.9) | 5,365 (8.0) | 119 (22.5) |
| Hx of stroke | 120 (0.1) | 14 (9.2) | 243 (0.4) | 33 (12.5) | 486 (0.7) | 90 (17.0) |
| AIS | 86 (0.0) | 13 (8.6) | 225 (0.3) | 32 (12.2) | 449 (0.7) | 84 (15.9) |
| ICH | 40 (0.0) | 4 (2.6) | 34 (0.1) | 6 (2.3) | 66 (0.1) | 15 (2.8) |

Abbreviations: CHF, congestive heart failure; CAD, coronary artery disease; HTN, hypertension; AF, atrial fibrillation; HLD, hyperlipidemia; AIS, acute ischemic stroke; ICH, intracerebral hemorrhage.

Note: Differences in characteristics between no stroke vs stroke groups were assessed using the chi-square tests for categorical variables and were all statistically significant (p<0.001).

**Supplemental Table 3. Characteristics of COVID-19 Patients with and without Stroke, Age ≥65 years**

|  | **Age 65-74** | | **Age 75-84** | | **Age 85 and older** | |
| --- | --- | --- | --- | --- | --- | --- |
|  | No Stroke  (n=40,430) | Stroke (n=735) | No Stroke  (n=22,349) | Stroke (n=681) | No Stroke  (n=11,429) | Stroke (n=393) |
| **Sex, n (%)** |  |  |  |  |  |  |
| Male | 20,205 (50.0) | 428 (58.2) | 10,820 (48.4) | 365 (53.6) | 4,564 (39.9) | 178 (45.3) |
| Female | 20,225 (50.0) | 307 (41.8) | 11,529 (51.6) | 316 (46.4) | 6,865 (60.1) | 215 (54.7) |
| **Comorbidities, n (%)** | |  |  |  |  |  |
| CHF | 4,476 (11.1) | 212 (28.8) | 4,377 (19.6) | 235 (34.5) | 3,261 (28.5) | 139 (35.4) |
| Hypertension | 23,718 (58.7) | 622 (84.6) | 15,703 (70.3) | 602 (88.4) | 8,359 (73.1) | 346 (88.0) |
| CAD | 7,354 (18.2) | 264 (35.9) | 6,474 (29.0) | 290 (42.6) | 3,497 (30.6) | 182 (46.3) |
| AF | 4,529 (11.2) | 211 (28.7) | 4,914 (22.0) | 272 (39.9) | 3,591 (31.4) | 193 (49.1) |
| Hyperlipidemia | 21,021 (52.0) | 533 (72.5) | 13,636 (61.0) | 522 (76.7) | 6,168 (54.0) | 277 (70.5) |
| Diabetes | 12,811 (31.7) | 414 (56.3) | 8,001 (35.8) | 347 (51.0) | 3,237 (28.3) | 133 (33.8) |
| Obesity | 9,946 (24.6) | 280 (38.1) | 4,727 (21.2) | 188 (27.6) | 1,340 (11.7) | 68 (17.3) |
| Smoking | 10,694 (26.5) | 338 (46.0) | 7,393 (33.1) | 323 (47.4) | 3,158 (27.6) | 133 (33.8) |
| Hx of smoking | 8,820 (21.8) | 271 (36.9) | 6,640 (29.7) | 288 (42.3) | 2,974 (26.0) | 129 (32.8) |
| Current | 2,951 (7.3) | 109 (14.8) | 1,275 (5.7) | 53 (7.8) | 309 (2.7) | 9 (2.3) |
| Hx of stroke | 584 (1.4) | 135 (18.4) | 548 (2.5) | 109 (16.0) | 357 (3.1) | 73 (18.6) |
| AIS | 547 (1.4) | 128 (17.4) | 511 (2.3) | 104 (15.3) | 333 (2.9) | 69 (17.6) |
| ICH | 74 (0.2) | 22 (3.0) | 66 (0.3) | 16 (2.3) | 47 (0.4) | 12 (3.1) |

Abbreviations: CHF, congestive heart failure; CAD, coronary artery disease; AF, atrial fibrillation; HLD, hyperlipidemia; AIS, acute ischemic stroke; ICH, intracerebral hemorrhage.

Note: Differences in characteristics between no stroke vs stroke groups were assessed using the chi-square tests for categorical variables and were all statistically significant (p<0.05) except current smoking in age 85 and older (p=0.62)
